# Immune System, Friend or Foe of Oncolytic Virotherapy?

**DOI:** 10.3389/fonc.2017.00106

**Published:** 2017-05-23

**Authors:** Anna C. Filley, Mahua Dey

**Affiliations:** ^1^Department of Neurosurgery, Indiana University–Purdue University Indianapolis, Indianapolis, IN, USA

**Keywords:** oncolytic virus, cancer, immunotherapy, innate immunity, adaptive immunity

## Abstract

Oncolytic viruses (OVs) are an emerging class of targeted anticancer therapies designed to selectively infect, replicate in, and lyse malignant cells without causing harm to normal, healthy tissues. In addition to direct oncolytic activity, OVs have shown dual promise as immunotherapeutic agents. The presence of viral infection and subsequently generated immunogenic tumor cell death trigger innate and adaptive immune responses that mediate further tumor destruction. However, antiviral immune responses can intrinsically limit OV infection, spread, and overall therapeutic efficacy. Host immune system can act both as a barrier as well as a facilitator and sometimes both at the same time based on the phase of viral infection. Thus, manipulating the host immune system to minimize antiviral responses and viral clearance while still promoting immune-mediated tumor destruction remains a key challenge facing oncolytic virotherapy. Recent clinical trials have established the safety, tolerability, and efficacy of virotherapies in the treatment of a variety of malignancies. Most notably, talimogene laherparepvec (T-VEC), a genetically engineered oncolytic herpesvirus-expressing granulocyte macrophage colony stimulating factor, was recently approved for the treatment of melanoma, representing the first OV to be approved by the FDA as an anticancer therapy in the US. This review discusses OVs and their antitumor properties, their complex interactions with the immune system, synergy between virotherapy and existing cancer treatments, and emerging strategies to augment the efficacy of OVs as anticancer therapies.

## Introduction

Oncolytic viruses (OVs) are viruses that selectively infect and kill malignant cells, leaving surrounding healthy cells unharmed. In addition to direct cytotoxic activity, OVs engage and amplify host immune responses, leading to the destruction of residual malignant cells and establishment of lasting antitumor immunity. Initial interest in the use of viruses to treat cancer dates back to observations made in the early 1900s of tumor regression in the context of natural viral infection (1). However, the feasibility of this approach was initially limited by viral pathogenicity and associated toxicity in human patients. Recent advances in genetic engineering technology enabling modifications that enhance the safety and efficacy of OVs spurred a renewed interest in oncolytic virotherapy (OVT). Improved tumor selectivity and inherent self-replication kinetics allow for targeted therapy and localized therapeutic amplification, reducing the risk of systemic toxicity.

Oncolytic viruses based on several different vectors including adenovirus, herpes simplex virus (HSV), vaccinia virus, Newcastle disease virus, measles virus, and reovirus have been shown to be tumor-specific, relatively non-toxic, and capable of inducing robust antitumor immune responses in animal models and human patients (2, 3). Talimogene laherparepvec (T-VEC), a genetically engineered HSV-based OV, was recently approved for the treatment of melanoma, representing the first oncolytic virus to be approved by the FDA as an anticancer therapy. Numerous clinical trials (Table 1) have demonstrated synergy between OVT and other standard and emerging anticancer therapies (4–14). Combination treatment, particularly with immune-modulating therapies, continues to be a promising field of research.

**Table 1:** **List of oncolytic viruses currently being tested in clinical trial**.

Virus	Name	Phase	Tumor	Combination	Reference
Adenovirus	ONYX-015	III	Squamous cell carcinoma head and neck (SCCHN)	Cisplatin	Khuri et al. (4)
		I/II	Pancreatic cancer	Gemcitabine	Hecht et al. (5)
		Pilot	Advancer cancers	Irinotecan + 5-FU or IL-2	Nemunaitis et al. (6)
		I/II	Advanced sarcoma	Mitomycin-C, doxorubicin, cisplatin	Galanis et al. (7)
	Oncorine (H101)	III	SCCHN or esophageal cancer	5-fluorouracil + cisplatin or adriamycin	Xia et al. (8)
	Ad5-CD/Tkrep	I	Prostate cancer	5-fluorocytosine, valganciclovir, radiation	Freytag et al. (9)
	ONCOS-201	I	Solid tumors	Cyclophosphamide	Ranki et al. (10)

Herpes simplex virus	Talimogene laherparepvec	I/II	SCCHN	Radiation, cisplatin	Harrington et al. (11)
		Ib	Melanoma	Ipilimumab (CTLA-4 inhibitor)	Puzanov et al. (12)
	G207	I	Glioma	Radiation	Markert et al. (13)

Reovirus	RT3D	I/II	Advanced cancers	Carboplatin/paclitaxel	Karapangiotou et al. (14)

Vaccinia	GL-ONC1	I	Head and neck carcinoma	Cisplatin, radiotherapy	NCT01584284
	JX-594 (Pexa-Vec)	I/IIa	Colorectal cancer	Irinotecan	NCT01394939

Activation of the host immune system is a crucial component of OV-mediated tumor destruction. However, immune responses can also prematurely terminate OV infection, precluding therapeutic efficacy. Optimization of viral replication and propagation as well as the generation of anticancer immunity remains a significant challenge facing OVT. With a better understanding of the complex immunological interactions between OVs, tumor cells, and the host immune system, the next generation of OVs will be poised to realize the full immunotherapeutic potential of OVT.

## Oncolytic Viruses

At the core of OVT is the natural propensity of viruses to infect malignant cells. This preference stems from an overlap between the cellular changes incurred during oncogenesis and those induced by viral infection. Cancer cells evolve to resist apoptosis and growth suppression, evade immune-mediated destruction, and proliferate indefinitely, characteristics also conducive to viral replication (15). Additionally, many tumors develop defects in cellular antiviral response pathways, like type I interferon (IFN) signaling, rendering them more susceptible to viral infection (16).

While some viruses, such as H1 autonomously replicating parvoviruses, reoviruses, Newcastle disease viruses (NDVs), vesicular stomatitis virus (VSV), mumps virus, etc., have a natural preference for infecting specific types of human tumor cells, others can be genetically modified to enhance tumor cell selectivity, including adenovirus, measles, vaccinia, and HSVs (3, 17). Various approaches have been explored for engineering the ideal oncolytic viral vector that will selectively target, infect, and destroy tumor cells, while sparing normal cells. Viral coat proteins can be altered to recognize specific tumor cell surface markers or utilize tumor-expressed proteases for cellular entry (3, 18). Genes necessary for viral replication can be placed under the control of tumor-specific promoters, or deleted entirely, rendering viral replication conditional upon genes constitutively active in malignant, but not normal, cells (3, 4).

## OVs and Tumor Microenvironment (TME)

### Virus-Mediated Tumor Cell Destruction

**Figure 1:**
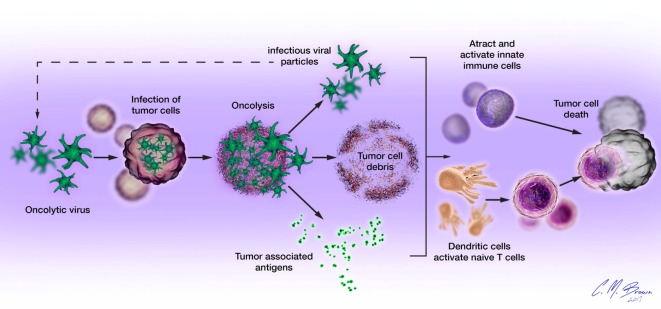
**Oncolytic viruses (OVs) mediate tumor cell destruction by two main mechanisms: (1) direct lysis of infected cells, OVs selectively infect malignant cells, hijacking their cellular transcription, and translation mechanisms in order to replicate**. Termination of the viral replication cycle induces tumor cell lysis and release of infectious viral progeny. Oncolysis also releases viral particles, tumor-associated antigens, and cellular damage-associated molecular patterns like calreticulin, heat shock proteins, and cellular ATP in a highly inflammatory process, termed “immunogenic cell death” and **(2) induction of host antitumor immune responses**. Cellular detection of viral infection and the products of oncolysis trigger the rapid activation of host antiviral responses and influx of immune cells that mediate the destruction of residual infected and uninfected tumor cells. The direct recognition and killing of tumor cells is primarily mediated by natural killer cells of the innate immune system and tumor antigen-specific CD8+ cytotoxic T lymphocytes of the adaptive immune system.

Oncolytic viruses mediate tumor cell death *via* direct and indirect mechanisms, functioning as both direct cytotoxic agents and therapeutic cancer vaccines (Figure 1). These mechanisms are connected by the propensity of many OVs to induce immunogenic forms of tumor cell death, including immunogenic apoptosis, necrosis, pyroptosis, and autophagic cell death, which activate host immune responses (19, 20). Immunogenic cell death (ICD) is characterized by cell surface exposure of calreticulin and heat shock proteins and the release of immune-stimulating molecules like ATP, uric acid, and high-mobility group box 1. Unlike normal apoptosis, which is mostly non-immunogenic and at time tolerogenic, ICD can induce antitumor immune response *via* dendritic cell (DC) activation. ICD of tumor cells also releases tumor-associated antigens (TAAs) that can be used to generate antigen-specific antitumor immunity (21–24).

### Native Antigen-Presenting Cells (APCs) and Viruses

Antigen presenting cells, such as DCs, are crucial mediators of innate and adaptive immunity, facilitating the generation of immune responses by releasing cytokines and activating naïve T cells. Recruited to sites of infection and inflammation, such as those induced by immunogenic tumor cell death, DCs capture viral and tumor antigens released during oncolysis and present them to naïve T cells, thereby initiating the generation of antigen-specific adaptive immune responses that mediate targeted destruction of residual and recurrent tumor cells (25).

### Tumor/Virus-Induced Cytokine Production

The TME is often characterized by a state of profound immunosuppression. Tumors overexpress cytokines like interleukin-10 and transforming growth factor-β (TGF-β), which inhibit natural antitumor immune responses. Tumor-derived cytokines and chemokines also include those promoting growth and vascularization like tumor necrosis factor-α (TNF-α) and vascular endothelial growth factor (25).

Viral infection stimulates the release of cytokines (IL-1, IL-6, IL-12, IL-18, IFN-γ, and TNF-α) and chemokines (RANTES, MIP-1α/β) from infected cells and resident and infiltrating immune cells, altering the balance of pro- and anti-inflammatory factors within the TME (26, 27). In addition to direct antiviral and immunoregulatory activities, these compounds mediate the recruitment of cytokine-releasing immune cells with additional effector functions. Viral infection and resulting localized inflammation enhance the effector functions of infiltrating immune cells, counteract tumor-induced immunosuppression, and facilitate the generation of antitumor immunity (27).

## Immunologic Barriers to Successful OVT

**Figure 2:**
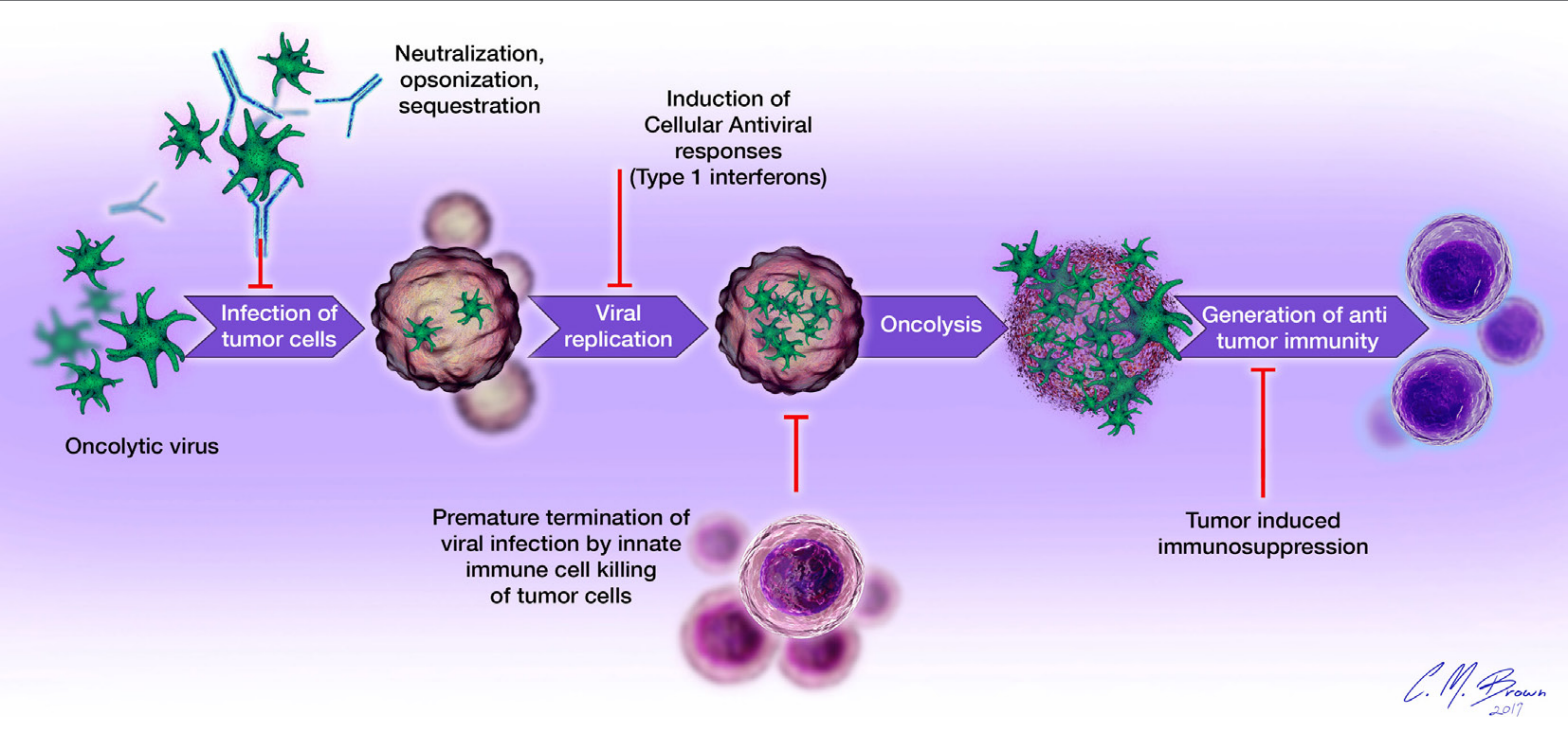
**Immunologic barriers to successful oncolytic virotherapy**: (1) oncolytic virus delivery to tumor sites is impeded by the presence of neutralizing antibodies, complement proteins, and sequestration in organs such as the liver and spleen; (2) cellular antiviral responses, such as type I interferon signaling limits viral replication within tumor cells; (3) destruction of infected tumor cells by cells of the innate immune system (neutrophils, macrophages, NK cells) prematurely terminates viral infection; (4) tumor-induced immunosuppression (elaboration of immunosuppressive cytokines, accumulation of regulatory T cells, overexpression of negative checkpoint regulators of T cell function) inhibits the generation and effector functions of antigen-specific antitumor immune responses.

Viral infection and oncolysis naturally activate innate and adaptive immune responses that are known to contribute to the killing of malignant cells. However, host immune responses to viral infection have also been shown to be detrimental to the overall efficacy of OVT. Numerous preclinical studies have demonstrated reduced viral replication, earlier clearance, and decreased antitumor efficacy in immunocompetent, compared to immunocompromised, hosts (2, 6, 28). Mechanisms of immunologic barriers to successful OVT are shown in Figure 2. The avidity and timing of oncolysis and activation of different components of the host immune response seem to play vital roles in determining the nature and extent of their relative contributions to the overall efficacy of OVT, with vector species and malignancy-specific differences (29–31).

## OVs and Innate Immunity

Detection of viral infection triggers the production of antiviral proteins, elaboration of cytokines, and recruitment of immune cells to the site of infection. Type I IFNs are antiviral proteins that reprogram gene expression in infected and uninfected cells to directly inhibit viral replication. IFNs also induce cell cycle arrest and apoptosis, upregulate major histocompatibility complex (MHC) expression, stimulate B cell immunoglobulin synthesis, and prompt the development and proliferation of memory T cells (27). Among first responders to viral infection are APCs and other innate immune cells, including neutrophils and NK cells (27, 32). In addition to the release of antiviral cytokines, these cells have unique mechanisms through which they can contribute to the antitumor efficacy of OVT. Neutrophils react to pathogens by secreting reactive oxygen species and proteases, inducing necrotic cell death and localized inflammation (4). In a heterotopic murine model of colon cancer, intratumoral neutrophil accumulation in response to OV infection resulted in tumor vasculature destruction and widespread tumor cell apoptosis (33). NK cells have also been shown to be key effectors of OV-induced antitumor immune responses (20, 23, 29). They specifically target cells lacking MHC molecules or displaying virally induced markers of cellular stress like MIC-A/B, inducing cell death by releasing granzyme and perforin enzymes, and activating apoptosis-inducing receptors (27, 28, 34). The agonist/antagonist relationship of the immune system and OV is not static but evolves with the phase of the infection and tumor destruction.

### Decreasing Virus Clearance

In order to exert maximal therapeutic effects, OVs must persist long enough and induce sufficient oncolysis to stimulate the generation of long-lasting adaptive antitumor immunity. However, viruses are foreign pathogens and naturally elicit host immune responses mediating their clearance. Upon introduction to the body, viral particles become coated with neutralizing antibodies and are eliminated in a complement-dependent fashion (35). Destruction of infected tumor cells by infiltrating innate immune cells and viral antigen-specific T cells can also terminate OV infection before full therapeutic effects have been achieved (33). Transient suppression of these early immune responses has potential to improve OV delivery to tumor sites, prolong viral infection, and enhance the overall therapeutic efficacy of OVT.

Inhibiting early intratumoral immune cell infiltration with low dose chemotherapy or TGF-β treatment has been shown to enhance viral replication, decrease clearance, and improve antitumor outcomes in several murine models of glioma (32, 34, 36). A recombinant VSV vector expressing a broad-spectrum chemokine-binding protein had similar effects, substantially prolonging the survival of animals with multifocal hepatocellular carcinoma (37).

Pretreatment with immunosuppressive chemotherapeutics like cyclophosphamide has been shown to improve viral delivery, promote replication, and enhance oncolytic activity of HSV-based OVs in murine models of glioma by depleting antiviral antibodies and impairing complement function (32, 38, 39) Viral coat modification through conjugation of polymers like polyethylene glycol and *N*-[2-hydroxypropyl]meth-acrylamide (HPMA) or lipid encapsulation can shield OVs from neutralizing serum factors and prevent the generation of new antiviral antibodies (2, 24). Alternatively, OVs can be hidden within carrier cell vectors and trafficked to tumor sites. In the context of malignant brain tumors, two cell types that have shown promising preclinical potential as OV carriers are mesenchymal stem cells (MSCs) (40) and neural stem cells (NSCs) (41). Both MSCs and NSCs possess a natural tropism for primary tumors and their metastases and are considered immune-privileged. MSCs have been studied extensively in preclinical settings (40, 42). In a small clinical trial of children with metastatic neuroblastomas refractory to frontline therapies, treatment with autologous MSCs carrying ICOVIR-5, an oncolytic adenovirus, was found to be safe and without significant systemic toxicity (43). For malignant glioma, NSC-based carriers not only improve the clinical efficacy of OV by protecting viruses from the host immune system but also through amplification of therapeutic payloads selectively at tumor sites (44, 45). In a comparison of MSCs and NSCs as cellular carriers for OVs, NSCs conferred a superior therapeutic efficacy in the context of malignant glioma (46). Based on these promising preclinical findings, the FDA recently approved the NSC HB1.F3-CD as a cell carrier carrying CRAd-S-pk7 OV for clinical trials in patients with newly diagnosed malignant glioma.

### OV Plus Chemotherapy

Preclinical and clinical studies have demonstrated significantly enhanced antitumor immune and clinical responses in patients receiving combination chemotherapy and OVT (4, 7–11, 14). The first such human clinical trial evaluated ONYX-015 (d11520), a genetically modified adenovirus, in combination with cisplatin and 5-fluorouracil in 37 patients with recurrent squamous cell head and neck cancer. Objective clinical responses were observed in 65% of treated patients, exceeding response rates seen with either agent alone (4). Chemotherapy complements virotherapy through a variety of known and unknown mechanisms, including the direct killing of malignant cells, enhancement of tumor cell immunogenicity, and suppression of antiviral immune responses (15, 47). Several chemotherapeutic agents, including oxaliplatin, doxorubicin, bleomycin, Bortezomib, and cyclophosphamide, have been shown to induce tumor ICD, promoting the generation of antitumor immune responses (19, 26, 48).

### OV Plus Immune Checkpoint Inhibitors

Destruction of malignant cells by the host immune system represents a crucial component of virotherapy. However, many tumors develop mechanisms to suppress the antitumor activity of incoming effector cells, for example by inducing overexpression of immune checkpoint regulators like CTLA-4 and PD-L1. T cell surface CTLA-4 competes with CD28 molecules for interaction with APC costimulatory molecules, transmitting inhibitory signals that suppress initial T cell activation. PD-L1, often overexpressed by tumor cells and tumor-infiltrating lymphocytes, binds PD-1 on activated T cells, inducing anergy or apoptosis (49, 50). Blockade of these molecules has been shown to improve T cell function and restore antitumor cellular immune responses. However, the clinical use of immune checkpoint inhibitors, particularly anti-CTLA-4 treatments, is limited by the high risk of associated severe autoimmune events resulting from systemic, uncontrolled T cell activation (50, 51). The unique ability of OVs to locally deliver and amplify therapeutic agents prompted an exploration of their use in combination with immune checkpoint inhibition. In a syngeneic murine model of malignant melanoma, the targeted, localized delivery of anti-CTLA-4 and anti-PD-L1 antibodies to the TME *via* an oncolytic measles virus induced comparably robust antigen-specific antitumor immune responses without evidence of immune-mediated toxicity (50). In another murine model of melanoma, intratumoral injection of combination NDV OV and anti-CTLA-4 antibody treatment resulted in regression of primary injected tumors and contralateral, untreated tumors, prolonged survival, and enhanced protection from tumor rechallenge as compared to treatment with either agent alone (49).

### OV Plus Histone Deacetylase (HDAC) Inhibitors

Histone deacetylase inhibitors are an emerging class of antineoplastic agents that enhance the therapeutic efficacy of OVT primarily by suppressing the induction of IFN-stimulated genes (16). HDAC inhibitors have been shown to augment viral replication, reduce early intratumoral immune cell recruitment, and enhance the oncolytic activity of OVs in a variety of tumor types (16). As epigenetic modifiers of transcription, HDAC inhibitors also shift cellular profiles of gene expression to favor the induction of growth arrest and apoptosis in cancer cells, antagonize tumor angiogenesis, and enhance tumor cell immunogenicity through increased expression of MIC-A/B, MHC, and costimulatory molecules (52).

### Increasing Antitumor Immune Response

Following initial OV-mediated tumor debulking, it is advantageous to promote host immune system-mediated destruction of any residual or recurrent malignant cells. This can be accomplished by mitigating tumor-induced immunosuppression, enhancing tumor cell immunogenicity, or directly activating host immune responses. Interventions that promote the development of localized inflammation can both counteract the immunosuppressive nature of the TME and recruit and activate effector immune cells. In a murine model of melanoma, OV expression of IL-12 and IL-18 increased intratumoral infiltration of activated NK cells, CD4+, and CD8+ T-cells, resulting in widespread tumor necrosis and prolonged survival (53). Combination treatment with other compounds that induce cellular stress or DNA damage, like chemotherapeutics, can enhance tumor immunogenicity by stimulating expression of NK cell-activating ligands and provoking tumor ICD (48). Increasing the availability of TAAs within the TME *via* induction of ICD or OV expression of specific TAAs can enhance antigen presentation and the generation of adaptive immunity. Incorporation of the ovalbumin protein within a VSV OV augmented the activation of ovalbumin-specific T cells, leading to increased antitumor effects in mice bearing B16ova tumors (54). Antigen-specific antitumor immune responses can be further enhanced by successive vaccination with two different TAA-expressing viruses, in which the second OV heightens the antitumor effects generated by the primary vaccination. This “prime-boost” method has been shown to induce durable adaptive immune responses that primarily target TAAs, rather than viral antigens (31, 55).

## OVs and Adaptive Immunity

Presentation of viral or TAA to cells of the adaptive immune system activates antigen-specific cellular and humoral immune responses. The primary antitumor effector cells of the adaptive immune system are CD8+ CTLs, which have been shown to be crucial mediators of OV-induced antitumor immunity, recognize specific antigens expressed on MHC class I molecules on the surface of infected and malignant cells and induce cell death through the release of perforin and granzymes. In the context of OVT, CTLs specific to viral antigens appear first, followed by development of TAA-specific CTLs (31). APCs also activate CD4+ T-helper cells, which release pro-inflammatory cytokines, promote CTL development, and are crucial in the development of antitumor immunity. Exposure to viral particles initiates humoral immune responses and the production of immunoglobulins from activated B cells. These neutralizing or opsonizing antibodies inhibit viral function and facilitate the clearance of viral infection (2, 28).

## Next-Generation Immune Modulating OVT

The host immune response to viral infection remains both an untapped resource and significant challenge facing OVT. The antitumor effects of OVs can theoretically be maximized by mitigating early immune responses to allow OV replication, oncolysis, and spread, followed by stimulation of the host immune system to destroy any residual tumor cells. Therapeutic manipulation of host immune responses represents a powerful strategy for optimizing both the oncolytic and immunotherapeutic potential of OVs. This can be achieved through combination therapy with chemotherapy, radiotherapy, immune checkpoint blockades, HDAC inhibitors, etc., or with single-agent OVs genetically engineered to express immune-modulating compounds.

In a murine model of colorectal cancer, OV expression of the chemokine RANTES (CCL5) prolonged the persistence of an oncolytic vaccinia virus, increased intratumoral lymphocyte infiltration, and enhanced antigen-specific antitumor responses, particularly in combination with DC-based immunotherapy (56). OV delivery of cytotoxic compounds or prodrug-activating enzymes can induce localized tumor damage without systemic side effects (56). AD5-CD/TKrep, an adenovirus expressing a cytosine deaminase/thymidine kinase (CD/TK) fusion protein that locally activates 5-fluorocytosine and ganciclovir pro-drugs, has been evaluated in two phase I clinical trials in patients with prostate cancer (9, 57).

## Future Direction

The future of OVT will focus on understanding and optimizing the complex interactions between OVs, tumor cells, and the host immune system. Elucidating relationships between factors such as patient immune status, malignancy type, tumor mutation profiles, and OV vector species, and patient responses to virotherapy will aid in the development of more efficacious, personalized treatments. Exploration of methods to improve OV access and delivery to tumor sites in terms of optimizing cell carrier-based delivery systems that maximize the therapeutic payload to the TME at the same time modulating host immune system are also promising areas of research. Overall, the most effective anticancer treatments will likely utilize a combination of therapies with different, synergistic mechanisms of tumor destruction.

## Conclusion

Oncolytic virotherapy is a novel approach to cancer treatment, uniquely combining direct cytotoxicity with antitumor immunotherapy. Clinical trials have established the safety and clinical efficacy of OVs, culminating in the recent FDA approval of the OV, T-VEC for treatment of malignant melanoma. An improved understanding of the relationships between OVs, tumors, and the host immune system will be necessary in the development of the next generation of OVs. Future OVs with improved tumor selectivity and cytotoxicity delivered in combination with immune-modulating therapies will significantly enhance the contribution of OVT to the field of oncology.

## Author Contributions

All authors listed, have made substantial, direct and intellectual contribution to the work, and approved it for publication.

## Conflict of Interest Statement

The authors declare that the research was conducted in the absence of any commercial or financial relationships that could be construed as a potential conflict of interest.
